# Involvement of APOE in Incidence of Revascularization in Patients Affected by Peripheral Arterial Disease: A Prospective Study from Southern Italy

**DOI:** 10.3390/jcm12165178

**Published:** 2023-08-09

**Authors:** Giuseppe Di Stolfo, Michele Antonio Pacilli, Davide Seripa, Giovanni De Luca, Maria Urbano, Carlo Coli, Carolina Gravina, Antonio Greco, Domenico Rosario Potenza, Mauro Pellegrino Salvatori, Gerit-Holger Schernthaner, Pavel Poredos, Mariella Catalano, Sandra Mastroianno

**Affiliations:** 1 Cardiovascular Department, Fondazione IRCCS Casa Sollievo della Sofferenza, 71013 San Giovanni Rotondo, Italy; 2 Complex Structure of Geriatrics, Medical Sciences Department, Fondazione IRCCS Casa Sollievodella Sofferenza, 71013 San Giovanni Rotondo, Italy; 3 Division of Angiology, Department of Internal Medicine 2, Medical University of Vienna, 1090 Vienna, Austria; 4 Department of Vascular Diseases, University Medical Center Ljubljana, 1000 Ljubljana, Slovenia; 5 Research Center on Vascular Diseases and Angiology Unit, University of Milan, L. Sacco Hospital, 20157 Milan, Italy

**Keywords:** apolipoprotein E, revascularization, peripheral arterial disease

## Abstract

Introduction. Atherosclerosis is a complex multifactorial disease and apolipoprotein E (APOE) polymorphism has been associated with cardiovascular events. The APOE gene, located on chromosome 19q13.2, has an important role in lipid metabolism, in particular on circulating cholesterol levels, implying further pleiotropic effects; from its polymorphism are derived three alleles (ε2, ε3 and ε4), which induce different phenotypes, while its impact on carotid and femoral atherosclerosis is still controversial. Objectives. The aim of the study is to investigate the relationship between APOE genotypes and peripheral revascularization in a cohort of patients affected by advanced peripheral arterial disease (PAD) at a prolonged follow-up. Materials and methods. Some 332 patients (259 males and 73 females; mean age 70.86 ± 7.95 years) with severe PAD were enrolled in a longitudinal study, with a 90.75 ± 32.25 month follow-up, assessing major adverse cardiovascular events (MACE). Results. As compared with ε3/ε3, in ε4 patients we observed a significant higher incidence of carotid (13.2% vs. 5.6%; HR = 2.485, 95% CI 1.062–5.814; *p* = 0.036) and lower limb (11.8% vs. 4.3%; HR = 2.765, 95% CI 1.091–7.008; *p* = 0.032) revascularizations and, accordingly, a higher incidence of total peripheral revascularizations (13.5% vs. 9.5%; HR = 2.705, 95% CI 1.420–5.151; *p* = 0.002). HR remained statistically significant even when adjusted for classic cardiovascular risk factors. Conclusions. In our observational study, we confirm that the ε4 allele is associated with higher total peripheral revascularization in patients with advanced atherosclerotic vascular disease at prolonged follow-up.

## 1. Introduction

Important manifestations of atherosclerotic disease, such as myocardial infarction and stroke, represent the leading cause of death in the world [1]. Chronic manifestations of atherosclerotic disease such as peripheral arterial disease (PAD), characterized by claudication and/or amputations, are due to steno-occlusion of the arteries of the lower limbs and have a great impact on quality of life and disability. Over 200 million people worldwide are affected [2]; it is the leading cause of lower limb amputation in the US and caused over 70 thousand deaths in 2017 [3]. PAD represents a rising global health and economic emergency, related to long-term care costs and population aging and spreading cardiovascular risk factors such as diabetes [4,5].

In recent decades, attention has increased towards the causes of atherosclerosis and its clinical consequences [6]. In addition to common cardiovascular risk factors such as diabetes, smoking, dyslipidemia and hypertension, it has been shown that numerous genetic components influence the process of atherosclerosis [7,8]. However, a better pathophysiologic model based on underlying genetic mechanisms needs to be implemented for adequate cardiovascular prevention and treatment under the umbrella of personalized medicine [9]. The APOE gene, located at chromosome 19q13.2, is one of the most studied; it has an important role in lipid metabolism, in particular on circulating cholesterol levels, implying further pleiotropic effects. From its polymorphism rise three alleles (ε2, ε3 and ε4) which form six different APOE genotypes and induce different plasma phenotypes. Alleles ε2 and ε4 are mainly associated with low-density lipoprotein-cholesterol (LDL-Ch), ε2 with lower levels andε4 with higher levels. In addition to being associated with increased lipoprotein remnants, the ε2 allele is more frequent in subjects with hypertriglyceridemia. The role of APOE polymorphisms on carotid and femoral atherosclerosisis still debated. Conversely, the ε4 allele appears to play a role in the development of type-2 diabetes mellitus and coronary artery disease [10,11]. Its pleiotropic effects involve oxidative processes, platelet aggregation, macrophage activation, central nervous system physiology, neuronal and glial cell homeostasis, adrenal response, inflammation and cell proliferation [12].

Atherosclerosis is the result of a low-grade inflammatory process that favors the deposition of inflammatory cells and cholesterol and the proliferation of smooth muscle cells in the intima [13]. A postprandial metabolism with the “postprandial oxidative stress” phenomenon induces impaired endothelial function and low-grade inflammation. Responses after a meal are variable in duration and extent and are regulated by physiological, dietary and genetic determinants, such as APOE gene polymorphisms. A greater inflammatory response was observed in ε4 carriers compared to ε3 overweight elderly people [14].

APOE polymorphism modulates susceptibility to many diseases, including neurodegenerative disorders (Alzheimer’s disease and cognitive decline) and atherosclerotic arterial disease [15]. The ε4 allele has proatherogenic effects but this simplification is reductive as environmental factors and additional genes could influence or modulate phenotypic expression [12]. This study represents the prolonged follow-up to our first observational registry [16], which was conducted to investigate a possible relationship between APOE gene polymorphisms and more aggressive forms of atherosclerotic disease among subjects with peripheral vascular disease.

## 2. Material and Methods

A cohort of cardiovascular controlled case series was followed up in a longitudinal study fulfilling the Declaration of Helsinki, the guidelines for Good Clinical Practice and Strengthening the Reporting of Observational Studies in Epidemiology (STROBE) [17]. 

The ethics committees on human experimentation of “Casa Sollievo della Sofferenza” Hospital (Approval Code TOMM40 version 11 Gen 13) approved the study protocol.

We identified 332 patients (78% males and 25% females) affected by peripheral arterial disease who were enrolled into the study from 1 November 2009 to 31 October 2017, after informed consent, using the following criteria: Caucasian race and advanced atherosclerosis defined as the presence of carotid plaque producing more than 50% stenosis by Doppler analysis velocimetry and/or Fontaine stage II or III claudication [16]. Exclusion criteria were carotid plaque with less than 50% stenosis, no claudication (Fontaine stage I), gangrene (Fontaine stage IV), active cancer with a life expectancy of less than six months and clinical diagnosis of Alzheimer’s disease. The presence of a cerebrovascular accident or peripheral revascularization that occurred during the previous eight weeks represented exclusion criteria.

Data about medical treatments, cancer, smoking, being overweight, dyslipidemia, arterial hypertension and type-2 diabetes mellitus, according to criteria of the World Health Organization and ATP III [18], were collected through interviews, clinical evaluations and reviews of medical records. Information on previous cerebrovascular accidents, myocardial infarctions, peripheral revascularizations (carotids and lower limbs) and myocardial revascularization procedures were also recorded. Patients underwent laboratory examination for blood chemistry and urine values for cholesterol, triglycerides, glucose, creatinine and microalbuminuria. All hematochemical and urinary tests were performed in the analysis laboratory of our institute. We evaluated insulin resistance by homeostatic model assessment (HOMA-IR) and we estimated glomerular filtration rate (eGFR) according to the modification of diet in renal disease study (MDRD) [19]. Body mass index (BMI) was estimated using the formula of weight divided by height squared (kg/m^2^). We measured brachial-ankle pulse wave (PWV) using an AngE system (Sonotechnik, Maria Rain, Austria), left ventricular ejection fraction (LVEF) and left ventricular mass with the Devereux formula by echocardiographic examination. Finally, we derived the ventricular mass index (LVMI) using left ventricular mass divided by body surface area. 

Echocardiographic examination and doppler ultrasonography of supra-aortic trunks, abdominal aorta and lower limb arteries were all performed, according to current guidelines [20], with the same ultrasound system and by two operators: a cardiologist dedicated to the study of heart and vessels and an angiologist dedicated to analysis of peripheral vessels.

We calculated PR, QRS and QTc intervals using a standard 12-lead electrocardiogram. We conducted a follow-up at 90.75 ± 32.25 months (range 1–124). During this period we recorded major adverse cardiovascular events (MACE), defined as cerebral ischemia, myocardial infarction, myocardial and/or peripheral revascularization, acute lower limb ischemia and cardiovascular death. We considered cardiovascular death as deaths occurring from heart failure, myocardial infarction, stroke, sudden death or ventricular arrhythmias. Furthermore, we recorded all cancer deaths. 

### 2.1. Genetic Analysis

Blood samples (2 mL) from each patient were collected in EDTA-containing tubes. We used standard methods to extract genomic DNA from peripheral blood and we identified in blinded fashion APOE genotypes [21,22]. Genetic examination was performed from 2010 to 2017 by two technicians and a geneticist belonging to the Complex Structure of Geriatrics of our center.

We observed the following genotype frequencies: 9.94% for ε2/ε3, 69.58% for ε3/ε3, 19.28% for ε3/ε4 and 1.20% for ε4/ε4. We did not identify the ε2/ε2 or ε2/ε4 genotypes. No differences were observed in respect to expected Hardy–Weinberg frequencies. Using these genotype frequencies found, we estimated allele frequencies: 4.97 for ε2, 84.19 for ε3 and 10.84 for ε4. Then, patients were grouped as follows: ε2 (ε2/ε3), ε3 (ε3/ε3) and ε4 (ε4/ε3 + ε4/ε4). We considered ε3/ε3 as a “wild-type”, being the most common genotype in the population, and patients with ε3/ε3 genotype as the reference group. 

### 2.2. Statistical Analysis

Continuous variables were presented as means ± standard deviation (SD); categorical variables were presented as frequency (%). In the groups, dichotomous variables were compared using the Pearson’s χ^2^ test. Hardy–Weinberg equilibrium was tested by a χ^2^ test. Quantitative data variables were compared using variance analysis (two-tailed unpaired *t*-test), after verifying normal distribution by a Kolmogorov–Smirnov test. *p* values < 0.05 were considered statistically significant. The Cox model was applied to estimate the cardiovascular event incidence by hazard ratio (HR) with 95% confidence interval (95% CI). HR was calculated crude and with adjustment for the common risk factors of BMI, hypertension, diabetes, dislipidemia, smoking, age, gender, LDL-cholesterol and triglycerides. Kaplan–Meier curves were used to show event-free rivascularization during follow-up. All statistical analyses were performed with SPSS 25.0 software (Chicago, IL, USA).

## 3. Results

### 3.1. Baseline Characteristics

We recruited 332 patients (259 males and 73 females; mean age 70.86 ± 7.95 years; range 45–88 years) affected by advanced PAD (52% with hemodynamic significant carotid stenosis, 34% with II or III stadium Fontaine and 14% suffering from both diseases). At baseline, 170 peripheral revascularization procedures had already been performed: in detail, 6 femoro-popliteal bypasses, 4 aorto-bifemoral bypasses, 1 ilaco-femoral thromboendarterectomy, 61 percutaneous transluminal angioplasties of the lower limb arteries, 47 carotid percutaneous transluminal angioplasties, 49 carotid thromboendarterectomies and 2 carotid bypasses. Moreover, 134 patients had a diagnosis of ischemic heart disease (54 with a history of myocardial infarction) and 124 had been revascularized through 41 surgical treatments, 72 endovascular treatments and 11 combined procedures. In addition, 52 patients had a history of previous cerebrovascular accidents or neuroradiological pictures of ischemic multifocal leukoencephalopathy.

Only 19% of collected patients were of normal weight (BMI between 20 and 25 kg/m^2^); 53% were overweight (BMI ≥ 25 kg/m^2^) and 28% were obese (BMI ≥ 30 kg/m^2^). Finally, 53 subjects had a personal history of cancer (with a life expectancy of more than 6 months).

We show clinical characteristics of the whole cohort grouped by APOE genotype in Table 1.

We observed a statistically significant difference of BMI: ε4 patients were less obese with respect to ε3 patients (BMI 27.07 ± 3.49 kg/m^2^ vs. 28.57 ± 4.22 kg/m^2^; *p* = 0.004). Compared to the wild type, despite being statistically non-significant, ε4 patients had higher fasting glucose levels (121.21 ± 47.88 mg/dL vs. 117.14 ± 35.94 mg/dL; *p* = 0.452).

Compared to ε3, we found statistical trends: ε2 patients were more obese (BMI 29.83 ± 3.50 kg/m^2^ vs. 28.57 ± 4.22 kg/m^2^; *p* = 0.108), more insulin resistant (HOMA-IR 9.70 ± 24.31 vs. 4.96 ± 7.59, *p* = 0.182), had higher triglyceride values (143.64 ± 75.20 mg/dL vs. 120.42 ± 56.60 mg/dL, *p* = 0.071) and had greater arterial stiffness as seen with PWV (16.89 ± 3.98 m/s vs. 14.77 ± 5.51 m/s; *p* = 0.152). However, in ε2 patients, with respect to ε3, we noticed no statistically significant differences in microalbuminuria (82.83 ± 152.58 µg/min vs. 71.49 ± 163.44 µg/min, *p* = 0.476), QTc interval (420.74 ± 26.75 ms vs. 415.12 ± 23.88 ms, *p* = 0.295), PR interval (169.60 ± 26.38 ms vs. 163.65 ± 27.41 ms, *p* = 0.35) and QRS duration (104.70 ± 24.16 ms vs. 100.26 ± 22.27 ms, *p* = 0.35). Compared to ε3 carriers, in ε2 carriers we found a trend in LVMI (82.84 ± 20.58 gr/m^2^ vs. 76.99 ± 20.12 gr/m^2^; *p* = 0.168) and no difference in LVEF (58.12 ± 5.86 ms vs. 58.58 ± 4.92 ms, *p* = 0.67). 

### 3.2. Comorbidities and Treatment

In Table 2 are reported the baseline most common comorbidities and medical treatments at the time of recruitment of the whole cohort and according to APOE genotype; no significant differences were observed between the different APOE carriers.

### 3.3. Outcomes

After a follow-up of 90.75 ± 32.25 months, we evaluated major cardiovascular accidents, arterial revascularization interventions and fatal events, as presented in Table 3. In total, 78 patients had at least one major adverse cardiovascular event (MACE).

We recorded nine surgical myocardial revascularizations and 31 percutaneous coronary interventions (24 with stenting and 7 with only drug balloon).

We observed revascularizations of the lower limbs performed in 2 cases by femoral-popliteal bypass surgery, in 4 cases with percutaneous transluminal balloon angioplasty and in 13 cases with percutaneous transluminal angioplasty plus stent insertion. In all patients, lower limb revascularization was performed to improve walking interval and reduce claudication. In 8 cases, a second attempt at revascularization was necessary. We did not note any lower limb amputations. 

On the other hand, analyzing carotid revascularizations, we counted 1 carotid-subclavian by-pass surgery, 10 endovascular treatments by transluminal angioplasty and stenting, and 15 thromboendarterectomies. Five patients with carotid stenosis were symptomatic just before undergoing revascularization (one stroke and four transitory ischemic attacks). In one patient we recorded a major complication during the first 24 h after carotid stenting: a hemorrhagic stroke. During the follow-up, we observed two significant carotid restenoses (≥70% evaluated by Doppler analysis), one intrastent and one at the anastomosis site of the carotid-subclavian bypass; both underwent a new revascularization treatment.

Comparison analysis between ε3 and ε4 carriers showed a greater number of MACE in ε4 carriers (25.0% vs. 23.8%; *p* = 0.744), even if not statistically significant.

During the follow-up, we observed that 42 patients underwent at least one peripheral revascularization. Prospective analysis showed that, with respect to ε3 carriers, ε4 carriers had a higher incidence of total peripheral revascularizations (13.5% vs. 9.5%; HR = 2.705, 95% CI 1.420–5.151; *p* = 0.002). In particular, ε4 carriers had a significantly higher incidence of carotid artery (13.2% vs. 5.6%; HR = 2.485, 95% CI 1.062–5.814; *p* = 0.036) and lower limb (11.8% vs. 4.3%; HR = 2.765, 95% CI 1.091–7.008; *p* = 0.032) revascularizations (Table 3). 

We calculated crude hazard ratio with adjustment for classical cardiovascular risk factors (age, gender, BMI, type-2 diabetes, hypertension, smoking and dyslipidemia) (Table 4). With respect to ε3, ε4 carriers have a higher risk of total peripheral revascularizations when these classic risk factors are considered (HR adjusted for age, gender, BMI, type-2 diabetes, hypertension, smoking and dyslipidemia = 2.730, 95% CI 1.404–5.309; *p* = 0.003) as resulting from eithercarotid (adjusted HR = 2.661, 95% CI 1.117–6.341; *p* = 0.027) or lower limb (adjusted HR = 2.810, 95% CI 1.044–7.569; *p* = 0.041) revascularization. 

The risk of ε4 carriers was higher for total peripheral revascularizations even when the variables LDL-cholesterol and triglycerides were considered (HR adjusted for LDL-cholesterol = 2.650, 95% CI 1.365–5.144; *p* = 0.004; HR adjusted for triglycerides = 2.589, 95% CI 1.337–5.049; *p* = 0.005).

**Figure 1:**
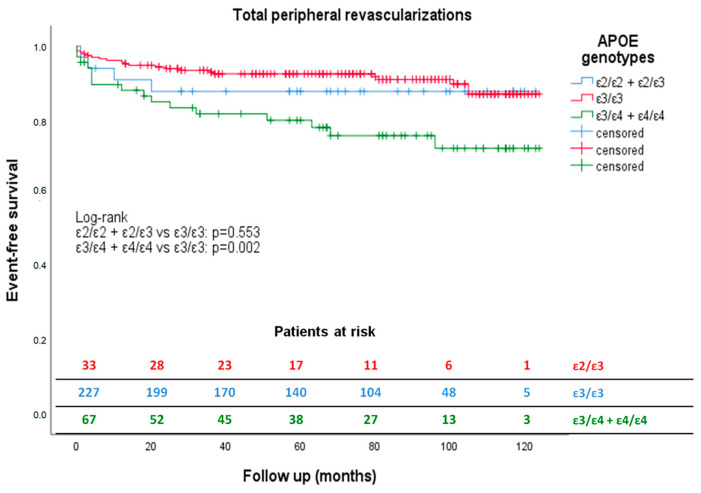
Kaplan–Meier estimates of total peripheral revascularizations according to APOE genotype during follow-up. Kaplan–Meier curves show how ε4 carriers (green) had a significantly reduced peripheral revascularization-free survival compared to ε3 carriers (red) during 90.75 ± 32.25 months’ follow-up. The ε2 patients (blue) did not have a peripheral revascularization-free survival statistically different from the ε3 carriers (red) during the same period.

Kaplan–Meier’s survival curves for revascularizations are shown in Figure 1.

During the follow-up we also recorded 85 deaths, including 28 by cancers, 24 by cardiovascular death and 33 by other causes. The fatal cardiovascular events that occurred were ten myocardial infarctions, seven strokes, one acute lower extremity ischemia, five advanced heart failure and one sudden death at age 79. The other major causes of death noted were sepsis (five cases), worsening of renal failure (three cases), exacerbation of respiratory failure (five cases), anemia, pneumonia, surgical complications, severe cognitive impairment and traumatic fracture. In any case, we did not find any significant difference based on APOE genotype.

In respect to ε3 carriers, we found a higher incidence, even if not statistically significant, of developing fatal events in ε2 carriers; in particular, cancer-related mortality was observed in ε2 carriers (15.2% vs. 7.4%; *p* = 0.107).

### 3.4. Subanalysis

We divided the cohort into two groups based on myocardial and/or peripheral revascularization at enrollment. We compared 217 patients with baseline revascularization versus 115 patients without baseline revascularization; the first group experienced more MACE (35% vs. 1.7%, *p* < 0.01) during follow-up (Table 5). In detail, patients already revascularized at baseline underwent myocardial (18.4%), carotid (7.8%) and lower limb (5.7%) revascularizations during follow-up. Conversely, no patients without baseline revascularization underwent treatment during follow-up.

Additionally, the revascularized group at baseline had a higher incidence of all-cause death (19.9% vs. 16.5%, *p* = 0.006); in detail, this group experienced 7.8% cardiovascular death and 10.1% cancer-related death, while the other group suffered 5.2% cardiac death and 5.2% cancer-related death.

Comparison analysis between ε2 and ε3 carriers showed no differences, irrespective of baseline revascularization status. In the group with baseline revascularization, we collected a statistically significant incidence of myocardial and peripheral revascularizations in ε4 carriers compared to ε3 (HR = 0.329, 95% CI 0.117–0.928; *p* = 0.036; HR = 2.521, % CI 1.322–4.008; *p* = 0.005, respectively). We calculated hazard ratio adjusted for previous cardiovascular risk factors (age, gender, BMI, type-2 diabetes, hypertension, smoking and dyslipidemia), confirming significant higher risk of peripheral revascularizations in ε4 carriers (HR = 2.011, 95% CI 1.121–3.607; *p* = 0.019). The statistically significant difference for myocardial revascularization was lost when we adjusted HR for diabetes. Graphical representation of peripheral revascularization-free survival curves in the group of patients with baseline revascularizations was similar to that shown in Figure 1. Log-rank was statistically significant for ε3 vs. ε4 carriers (*p* = 0.004) and not significant for ε3 vs. ε2 carriers (*p* = 0.469).

## 4. Discussion

PAD is a complex disease that recognizes several cardiovascular risk factors such as age, smoking, diabetes mellitus, hypertension, dyslipidaemia, environmental factors and genetic predisposition. Numerous research groups have investigated genes that may play a role in initiation and progression of atherosclerosis and predisposition to more aggressive outcomes using several genomic approaches such as linkage, genome-wide association (GWAS) and candidate gene evaluation studies [7]. It is well accepted that genetic factors are associated with peripheral artery disease, and APOE polymorphism plays a role in this issue, partially in relation to lipid levels [23]. Multiple studies have investigated this association with inconsistent results and mixed interpretations, suggesting a variable role of APOE ε4 polymorphism on MACE in different populations [24,25]. Conflicting results are due to different study designs, geographic and ethnic origin of cohorts, allelic frequency, and potential gene–gene and gene–environment interaction [26]. In addition to atherosclerotic disease, APOE polymorphisms have been studied as factors associated with various pathological conditions such as neurodegenerative pathologies [15], diabetes [27], kidney disease [28], worse cognitive ability [29], stroke [30] and cancer [31]. A meta-analysis published in 2016 evaluated the association of polymorphisms of the APOE gene with susceptibility to atherosclerosis; a subgroup examination based on clinical phenotypes showed that ε4 carriers were prone to develop major clinical manifestations related to atherosclerosis [32]. Our study analyzed the correlation between APOE genotype and the incidence of aggressive manifestations of atherosclerotic disease as cardiovascular death, myocardial infarction, cerebral ischemia, surgical or endovascular revascularization of coronary, and/or peripheral arteries and acute lower extremity ischemia in 332 patients. Our cohort is characterized by elderly subjects coming from Southern Italy affected by peripheral vascular disease and previous cardiovascular events (69.6%) such as ischemic heart disease, cerebrovascular accident and arterial reperfusion and presenting common risk factors already managed by a tailored therapeutic approach. As already observed in the small cohort of patients in the previous prospective observational study of 31.65 ± 21.11 months [16], a longer observation period (90.75 ± 32.25 months) of follow-up confirmed that ε4 carriers have an increased risk of cardiovascular events, in particular of carotid and lower limb revascularizations. Furthermore, subanalysis based on previous revascularization showed that the revascularized group was at very high risk of subsequent major cardiovascular events and the ε4 genotype further stratifies higher risk patients. 

Differing results reported in the literature may be related to diverse outcomes and populations analyzed for both age and stage of disease, as hypothesized for the Secondary Manifestations of ARTerial disease (SMART) study. This study analyzed 7418 subjects, from The Netherlands, aged 56.7 ± 12.4 years (72% with cardiovascular disease and 28% with only classical risk factors), highlighting more events and peripheral reperfusions in ε2 carriers compared with ε3 [24]. On the contrary, our study analyzed very-high-risk elderly subjects (mean age of 70.86 ± 7.95 years) coming from a limited geographic area and affected by advanced manifestation of atherosclerosis at the time of recruitment. The cohort selected with these characteristics showed an incidence of 12.7% of new peripheral revascularizations. This is different from the SMART study, where an incidence of 6.1% of new peripheral artery disease events was observed despite a longer median follow-up time (8.1 years). A plausible explanation for the divergence of our results could be secondary not only to the different ages and degree of disease analyzed but also to different ethnic origins and consequent emerging linkage imbalances [33]. 

Even in the Study of Health in Pomerania (SHIP), conducted in Germany on 3327 participants aged between 20 and 79 years, ε2 carriers were more predisposed to cardiovascular events (myocardial infarction) and peripheral atherosclerosis assessed by measuring thickening intimal carotid. The ε4 allele had no effect on carotid intimal thickening, carotid plaque progression, myocardial infarction or stroke [34]. We hypothesize that the different result was due to several cardiovascular risk factors in our patients, as the SHIP study was conducted on subjects randomized from the general population at lower cardiovascular risk.

Conversely, when a cohort with known carotid atheroma is selected, as in a recent case–control study conducted in northwest China, the role of APOE genotypes in the progression of artery atherosclerosisis is more evident in ε4 carriers [35].

An evaluation of APOE polymorphism and carotid atherosclerosis in Korean subjects, characterized by being over the age of 45 years, rural area origin, mostly female gender and not affected by peripheral atherosclerosis, showed that ε4 carriers had a higher risk of carotid plaque, and this result was mediated by lipids [36].

In the Three-City (3C) study, a longitudinal study conducted on 5856 elderly subjects recruited from three French cities, carotid plaques were more present in ε4 carriers compared to ε3 homozygous carriers [37]. The correlation between carotid atherosclerosis and APOE polymorphism was independent of lipid levels, according to our observation on ε4 carriers, with doubled risk compared to ε3 carriers (HR = 2.661, 95% CI 1.117–6.341; *p* = 0.027) of developing critical carotid plaque requiring revascularization independent of common vascular risk factors.

Recent clinical studies analyzed the association between APOE polymorphism and incidence of in-stent restenosis after vertebral and carotid artery stenting, demonstrating that ε4 polymorphism is an independent risk factor for restenosis [38,39].

According to the literature, we report a strong association between insulin resistance, hypertrigliceridemia and ε2 polymorphism, such as direct correlation between insulin resistance and arterial stiffness [40]; increased QTc duration, cardiac hypertrophy and microalbuminuria were the results of vascular aging and worse ventriculo-arterial coupling in this group [41,42].

On the basis of our results, we strongly believe that in the future APOE polymorphism needs to be included in complex genetic risk scores to assess cardiovascular risk, even in patients affected by advanced peripheral vascular disease. Introduction of new lipid lowering drugs, such as PCSK9 inhibitors, bempedoic acid and incliserin, will lead to better treatment and outcomes in patients affected by advanced atherosclerosis; however, the literature underlines the strict relationship between PCSK9 and APOE in both lipid profile and vascular disease [43,44]. In the future, we need to discern if the therapeutic effect might differ among patients with different genetic profiles, suggesting a more aggressive treatment in special subgroups following precision medicine indications.

An approach to patients affected by peripheral artery disease needs to be completed, including different dimensions from classic risk factor profiles to more advanced genetic analysis, taking into account the role of APOEε4 for atherosclerosis development in different vascular beds.

Our study presents several limitations, mainly represented by population size, a single center, observational approach, narrow regional origin of enrolled patients, Caucasian ethnicity, gender bias and a lack of apolipoprotein level dosage.

## 5. Conclusions

In our observational study, we confirm that the ε4 allele is associated with a higher incidence of aggressive events of cardiovascular disease, in particular of peripheral revascularization (carotid and lower limb revascularization) in patients with advanced atherosclerotic vascular disease at prolonged follow-up, underlying the need for a strict periodical clinical reassessment in specific subgroups, even in the elderly population, in the setting of a personalized medicine approach.

## Figures and Tables

**Table 1:** Description of clinical characteristics at baseline of the whole cohort, grouped by APOE genotype.

	All Patients			APOE Genotypes		
		ε2/ε3	*p*	ε3/ε3	*p*	ε3/ε4 + ε4/ε4
Number of subjects	332	33		231		68
Age (years)	70.86 ± 7.95	71.70 ± 8.49	0.53	70.79 ± 7.71	0.91	70.68 ± 8.56
Gender (male/female)	259/73	23/10	0.19	184/47	0.57	52/16
BMI (kg/m^2^) ^a^	28.38 ± 4.08	29.83 ± 3.50	0.10	28.57 ± 4.22	0.004	27.07 ± 3.49
Waist circumference (cm)	100.73 ± 10.54	102.73 ± 8.92	0.42	101.18 ± 10.81	0.08	98.15 ± 12.41
Waist–hip ratio	0.96 ± 0.07	0.98 ± 0.06	0.18	0.96 ± 0.06	0.92	0.96 ± 0.09
Systolic blood pressure (mmHg)	133.64 ± 17.94	133.37 ± 15.21	0.90	132.91 ± 17.73	0.23	136.30 ± 19.91
Diastolic blood pressure (mmHg)	79.52 ± 6.50	80.67 ± 7.20	0.24	79.10 ± 6.24	0.20	80.38 ± 6.99
Pulse pressure (mmHg)	54.12 ± 15.68	52.69 ± 13.77	0.73	53.81 ± 15.70	0.40	55.91 ± 16.62
Fasting glucose (mg/dL)	118.37 ± 39.44	120.91 ± 43.80	0.58	117.14 ± 35.94	0.45	121.21 ± 47.88
HOMA-IR	5.17 ± 10.02	9.70 ± 24.31	0.18	4.96 ± 7.59	0.33	4.12 ± 5.13
Triglycerides (mg/dL)	120.74 ± 56.75	143.64 ± 75.20	0.07	120.42 ± 56.60	0.16	110.54 ± 42.77
Total Ch (mg/dL)	166.37 ± 40.77	164.18 ± 53.16	0.82	166.34 ± 39.04	0.82	167.57 ± 40.15
HDL-Ch (mg/dL)	48.61 ± 12.36	49.21 ± 11.91	0.72	48.40 ± 12.50	0.73	49.00 ± 12.26
LDL-Ch (mg/dL)	94.46 ± 35.29	86.87 ± 47.61	0.22	94.93 ± 32.99	0.72	96.60 ± 35.86
Serum creatine (mg/dL)	1.05 ± 0.57	1.03 ± 0.37	0.79	1.06 ± 0.62	0.80	1.04 ± 0.43
eGFR (mL/min/1.73 m^2^)	81.79 ± 28.41	79.54 ± 23.51	0.63	82.40 ± 29.778	0.68	80.92 ± 25.53
Microalbuminuria (µg/min)	70.14 ± 159.97	82.85 ± 152.58	0.72	71.49 ± 163.44	0.60	71.49 ± 163.44
PWV (m/s)	14.66 ± 5.42	16.89 ± 3.98	0.18	14.77 ± 5.51	0.23	13.27 ± 5.36
PR interval (ms)	163.93 ± 27.51	169.60 ± 26.38	0.35	163.65 ± 27.41	0.81	162.57 ± 28.66
QRS interval (ms)	100.73 ± 21.51	104.70 ± 24.16	0.35	100.26 ± 22.27	0.89	100.69 ± 21.43
QTc interval (ms)	415.34 ± 24.12	420.74 ± 26.75	0.29	415.12 ± 23.91	0.69	413.61 ± 23.88
Heart rate (bpm)	70.08 ± 10.67	73.10 ± 8.53	0.14	70.12 ± 10.76	0.30	68.53 ± 11.13
LVEF (%)	58.30 ± 5.80	58.58 ± 4.92	0.67	58.12 ± 5.86	0.42	58.79 ± 6.09
LVMI (gr/m^2^)	76.83 ± 19.97	82.84 ± 20.58	0.16	76.99 ± 20.12	0.28	73.46 ± 18.81

^a^ statistically significant; BMI: body mass index; eGFR: estimated Glomerular Filtration Rate; LVEF: left ventricular ejection fraction; LVMI: left ventricular mass index; PWV: pulse wave velocity.

**Table 2:** Description of comorbidities and medical treatments at baseline of the whole cohort, grouped by APOE genotype.

	All Patients		APOE Genotypes							
			ε2/ε3		*p*	ε3/ε3		*p*	ε3/ε4 + ε4/ε4	
Comorbidities										
Hypertension	294	(88.6%)	28	(84.8%)	0.51	205	(88.7%)	0.82	61	(89.7%)
Dyslipidemia	270	(82.3%)	29	(87.9%)	0.43	187	(82.4%)	0.57	54	(79.4%)
Type-2 diabetes	156	(47.0%)	16	(48.5%)	0.85	108	(46.8%)	0.96	32	(47.1%)
Smoking	75	(22.6%)	7	(21.2%)	0.78	54	(23.4%)	0.63	14	(20.6%)
Myocardial infarction	134	(40.4%)	10	(30.3%)	0.25	94	(40.7%)	0.61	30	(44.1%)
Stroke	52	(15.7%)	4	(12.1%)	0.45	40	(17.3%)	0.27	8	(11.8%)
Carotid revascularization	98	(29.5%)	12	(36.4%)	0.30	64	(27.7%)	0.45	22	(32.4%)
Lower limb revascularization	72	(21.7%)	5	(15.2%)	0.41	49	(21.2%)	0.36	18	(26.5%)
Myocardial revascularization	124	(37.5%)	9	(27.3%)	0.24	87	(37.8%)	0.60	28	(41.2%)
Cancer	53	(16%)	4	(12.1%)	0.39	42	(18.2%)	0.12	7	(10.3%)
Medical treatments										
ARBs	134	(40.4%)	14	(42.4%)	0.63	88	(38.1%)	0.18	32	(47.1%)
ACE inhibitors	124	(37.3%)	9	(27.3%)	0.12	95	(41.1%)	0.08	20	(29.4%)
Calcium channel blockers	99	(29.8%)	13	(39.4%)	0.15	63	(27.3%)	0.29	23	(33.8%)
β-blockers	90	(27.1%)	6	(18.2%)	0.26	63	(27.3%)	0.56	21	(30.1%)
Diuretics	149	(44.9%)	20	(60.6%)	0.07	102	(44.2%)	0.51	27	(39.7%)
Antiplatelet	291	(87.7%)	28	(84.8%)	0.41	207	(89.6%)	0.10	56	(82.4%)
Lipid-lowering drug	288	(86.7%)	29	(87.9%)	0.94	202	(87.4%)	0.44	57	(83.8%)
Antidiabetic therapy	111	(33.4%)	11	(33.3%)	0.96	76	(32.9%)	071	24	(35.3%)

Data are presented as number (%) of subjects. ACE inhibitors: angiotensin-converting enzyme inhibitors; ARBs: angiotensin receptor blockers.

**Table 3:** Cardiovascular events, revascularizations and fatal events at follow-up of the whole cohort, grouped by APOE genotype. Hazard ratios for events according to APOE genotype.

			APOE Genotypes									
	All Patients		ε2/ε3				ε3/ε3				ε3/ε4 + ε4/ε4	
					*p*	HR (95% CI)			*p*	HR (95% CI)		
Cardiovascular events												
MACE	78	(23.5%)	2	(18.2%)	0.588	1.262 (0.543–2.931)	55	(23.8%)	0.744	1.095 (0.635–1.886)	17	(25.0%)
Revascularizations												
Myocardial	40	(12.0%)	2	(6.1%)	0.223	2.425 (0.583–10.095)	34	(14.7%)	0.074	0.389 (0.138–1.096)	4	(5.9%)
Carotid	26	(7.8%)	4	(12.1%)	0.153	0.442 (0.144–1.356)	13	(5.6%)	0.036	2.485 (1.062–5.814)	9	(13.2%)
Lower limb	19	(5.7%)	1	(3.0%)	0.816	1.276 (0.163–9.986)	10	(4.3%)	0.032	2.765 (1.091–7.008)	8	(11.8%)
Total peripheral	42	(12.7%)	4	(12.1%)	0.555	0.725 (0.250–2.106)	22	(9.5%)	0.002	2.705 (1.420–5.151)	16	(13.5%)
Fatal events												
Total death	85	(25.6%)	12	(36.4%)	0.127	0.616 (0.331–1.147)	58	(25.1%)	0.609	0.862 (0.489–1.521)	15	(22.1%)
Cardiovasculardeath	24	(7.2%)	2	(6.1%)	0.992	1.008 (0.232–4.388)	17	(7.4%)	0.927	1.048 (0.384–2.862)	5	(7.4%)
Cancer death	28	(8.4%)	5	(15.2%)	0.107	0.440 (0.162–1.195)	17	(7.4%)	0.738	1.172 (0.462–2.974)	6	(8.8%)

Data are presented as number (%) of subjects. HR: hazard ratio. MACE: major adverse cardiovascular events.

**Table 4:** Hazard ratio adjustment for classical risk factors in ε4 carriers with respect to ε3 carriers.

Revascularizations								
	Myocardial		Carotid		Lower Limb		Total Peripheral	
HR Adjustment (95% CI), *p*								
Age and gender	0.390 (0.138–1.099)	0.075	2.523 (1.081–5.934)	0.032	2.684 (1.057–6.814)	0.038	2.719 (1.426–5.185)	0.002
BMI	0.380 (0.134–1.080)	0.069	2.523 (1.062–5.994)	0.036	2.744 (1.059–7.109)	0.038	2.705 (1.402–5.220)	0.003
Diabetes	0.390 (0.138–1.099)	0.075	2.492 (1.065–5.830)	0.035	2.924 (1.153–7.415)	0.024	2.720 (1.428–5.180)	0.002
Hypertension	0.385 (0.137–1.087)	0.071	2.467 (1.054–5.773)	0.037	2.781 (1.097–7.049)	0.031	2.703 (1.419–5.148)	0.002
Smoking	0.390 (0.138–1.100)	0.075	2.487 (1.062–5.823)	0.036	2.838 (1.119–7.196)	0.028	2.721 (1.428–5.185)	0.002
Dyslipidemia	0.396 (0.140–1.118)	0.080	2.514 (1.074–5.883)	0.034	2.724 (1.074–6.905)	0.035	2.700 (1.418–5.143)	0.003
LDL-Ch	0.397 (0.141–1.121)	0.081	2.166 (0.897–5.228)	0.086	2.720 (1.012–7.311)	0.047	2.650 (1.365–5.144)	0.004
Triglyceride	0.401 (0.142–1.134)	0.085	2.212 (0.914–5.353)	0.078	2.611 (0.971–7.027)	0.057	2.589 (1.337–5.049)	0.005
Age, gender, BMI, diabetes, hypertension, smoking and dyslipidemia	0.364 (0.127–10.45)	0.060	2.661 (1.117–6.341)	0.027	2.810 (1.044–7.569)	0.041	2.730 (1.404–5.309)	0.003

**Table 5:** Cardiovascular events, revascularizations and fatal events at follow-up of the whole cohort divided by baseline revascularization and grouped by APOE genotype. Hazard ratios for events according to APOE genotype.

			APOE Genotypes									
	Patients		ε2/ε3				ε3/ε3				ε3/ε4 + ε4/ε4	
					*p*	HR (95% CI)			*p*	HR (95% CI)		
Revascularized at baseline												
Cardiovascular events												
MACE	76	(35.0%)	6	(30.0%)	0.262	2.263 (0.543–9.419)	53	(35.8%)	0.99	1.004 (0.581–1.734)	17	(34.7%)
Revascularizations												
Myocardial	40	(18.4%)	2	(10.0%)	0.26	2.263 (0.543–9.419)	34	(23.0%)	0.036	0.329 (0.117–0.928)	4	(8.2%)
Carotid	26	(7.8%)	4	(20.0%)	0.12	0.413 (0.134–1.266)	13	(8.8%)	0.05	2.529 (0.998–6.410)	9	(18.4%)
Lower limb	19	(5.7%)	1	(5.0%)	0.87	0.193 (0.152–9.336)	10	(6.8%)	0.06	2.232 (0.953–5.227)	8	(16.3%)
Total peripheral	42	(12.7%)	4	(20.0%)	0.47	0.677 (0.233–1.966)	22	(14.9%)	0.005	2.521 (1.322–4.008)	16	(32.7%)
Fatal events												
Total death	66	(19.9%)	10	(50%)	0.05	0.501 (0.253–1.002)	44	(29.7%)	0.56	0.829 (0.438–1.569)	12	(24.5%)
Cardiovascular death	17	(7.8%)	2	(10.0%)	0.53	0.615 (0.136–2.782)	11	(7.4%)	0.89	1.084 (0.345–3.405)	4	(8.2%)
Cancer death	22	(10.1%)	3	(15.0%)	0.44	–	14	(9.5%)	0.81	1.321 (0.137–12.71)	5	(10.2%)
Non-revascularized at baseline												
Cardiovascular events												
MACE	2	(1.7%)	0	(0%)	0.57	–	2	(2.4%)	0.49	–	0	(0%)
Revascularizations												
Myocardial	0	(0%)	0	(0%)	–	–	0	(0%)	–	–	0	(0%)
Carotid	0	(0%)	0	(0%)	–	–	0	(0%)	–	–	0	(0%)
Lower limb	0	(0%)	0	(0%)	–	–	0	(0%)	–	–	0	(0%)
Total peripheral	0	(0%)	0	(0%)	–	–	0	(0%)	–	–	0	(0%)
Fatal events												
Total death	19	(16.5%)	2	(15.4%)	0.95	1.049 (0.238–4.621)	14	(16.9%)	0.82	0.862 (0.247–3.007)	3	(16.5%)
Cardiovascular death	6	(5.2%)	0	(0%)	0.58	–	5	(6.0%)	0.89	0.86 (0.100–7.361)	1	(5.3%)
Cancer death	6	(5.2%)	0	(0%)	0.36	–	5	(6.0%)	0.89	1.073 (0.387–2.980)	1	(5.3%)

Data are presented as number (%) of subjects. HR: hazard ratio. MACE: major adverse cardiovascular events.

## Data Availability

The data presented in this study are available on request from the corresponding author. The data are not publicly available due to privacy issue.
